# Circular RNA-9119 suppresses in ovarian cancer cell viability via targeting the microRNA-21-5p–PTEN–Akt pathway

**DOI:** 10.18632/aging.103470

**Published:** 2020-07-16

**Authors:** Jianming Gong, Xiaoyang Xu, Xuanli Zhang, Yingqiao Zhou

**Affiliations:** 1 Obstetrics and Gynecology Department, The First Affiliated Hospital of Wenzhou Medical University, Wenzhou 325000, Zhejiang, China

**Keywords:** ovarian cancer, circ9119, miR-21, PTEN, apoptosis

## Abstract

We aimed to assess the regulatory role of circular RNA (circRNA)-9119 (circ9119) in ovarian cancer (OC) cell viability. The expression of circ9119 was clearly reduced in OC tissues and cell lines, whereas the microRNA-21-5p (miR-21) levels were elevated compared with those in normal healthy control tissues and immortalized fallopian epithelial cell line FTE187. Further, circ9119 was overexpressed, causing a notable decrease in the viability and proliferation of OC cells and an increase in apoptosis. Further study showed that circ9119 upregulation resulted in a decrease in miR-21 levels. Bioinformatics forecasting (starBase and TargetScan) and dual luciferase reporter assay demonstrated that circ9119 acts as an miR-21 sponge. Recovery of miR-21 expression in circ9119-overexpressing OC cells showed that miR-21 exhibited the opposite effect on circ9119; moreover, its recovery could suppress the effects of circ9119 overexpression, recover cell proliferation, and reduce apoptosis. Furthermore, miR-21 was found to target phosphatase and tensin homologue (PTEN) 3′ untranslated region. PTEN protein and mRNA expression was reduced in OC tissues and cells, whereas it was increased on transfection with an miR-21 inhibitor. Thus, circ9119 could regulate cell proliferation and apoptosis of OC cells via by acting as an miR-21 sponge and targeting the PTEN–Akt pathway.

## INTRODUCTION

Ovarian cancer (OC) is a common gynecologic disease worldwide. In 2018, approximately 300,000 patients developed OC worldwide and approximately 180,000 OC-related deaths were reported [1]. Despite advancements in routine treatments, the mortality rate remains high because of unsatisfactory prognosis and recurrence [2].

Circular RNAs (circRNAs) have displayed potential as gene modulators in recent years because they comprise microRNA (miRNA) response elements and struggle with mRNAs [3]. Thus, circRNAs can act as molecular sponges for miRNAs and eventually inhibit miRNA objective genes and affect posttranscriptional regulation [4]. Recently, several circRNAs have been recognized as latent biomarkers for OC diagnosis and prognosis [5]. Because circRNAs exert their effects via various cellular and molecular mechanisms associated with cancer occurrence, particularly via regulation of cell proliferation, it has been shown that the downregulation of these molecules is associated with OC occurrence and progression [6]. Therefore, the function and roles of OC-specific circRNAs must be explored to enhance our understanding of OC and provide new opportunities for developing more favorable treatment or diagnosis methods.

Therefore, the present study aimed to evaluate the role and function of circRNAs in OC. To investigate the functions of circRNA and miRNA in OC progression, we evaluated the expression profiles of circRNAs in OC samples and normal healthy ovarian tissue as well as in ovarian cancer cell lines and normal cells. Moreover, the correlations of circRNA with miRNA-21-5p (miR-21) and the phosphatase and tensin homologue (PTEN)–Akt pathway were explored.

## RESULTS

### Decreased circ-9119 levels in OC samples and cells

**Figure 1:**
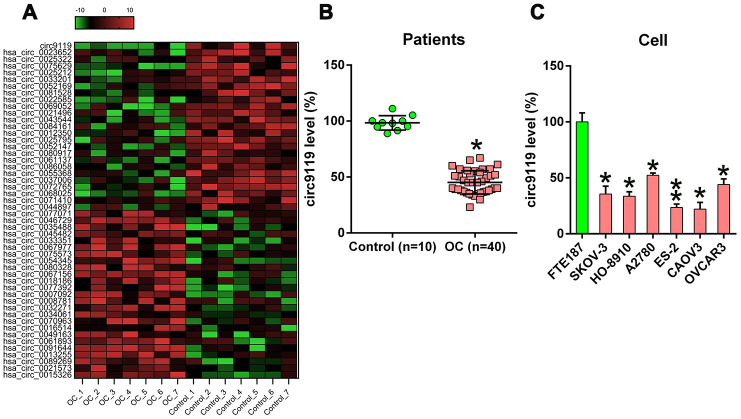
**cicr9119 expression in OC samples and cell lines.** (**A**) Hierarchical clustering evaluation revealed changes in the circRNA expression patterns in OC (n = 7) and control groups (n = 7); each group comprised seven samples (>2 × expression distinction; P < 0.05). Expression values are noted in different colors reflecting different levels. (**B**) qRT–PCR showed circ9119 expression in OC samples (n = 40) and normal healthy control samples (n = 10). (**C**) qRT–PCR showed circ9119 expression in OC and FTE187 cells. *P < 0.05, **P < 0.01 vs. specific groups.

circRNA expression was evaluated in seven OC samples and seven control samples with hierarchical clustering (Figure 1A). A total of 50 circRNAs displayed marked expression patterns in OC samples compared with those in control samples. Among these, 25 circRNAs displayed decreased expression and 25 displayed increased expression in the OC samples.

Among them, circ9119 displayed the most significant change in expression. Further, the qRT–PCR results of circRNA expression assessed in 40 OC samples and 10 control samples showed that compared with the control samples, the OC tissues exhibited a remarkable decrease in circ9119 expression (Figure 1B). Furthermore, all the six OC cell lines displayed reduced circ9119 expression compared with FTE187 (Figure 1C).

### Circ-9119 overexpression suppressed ES-2 and SKOV-3 cell viability

**Figure 2:**
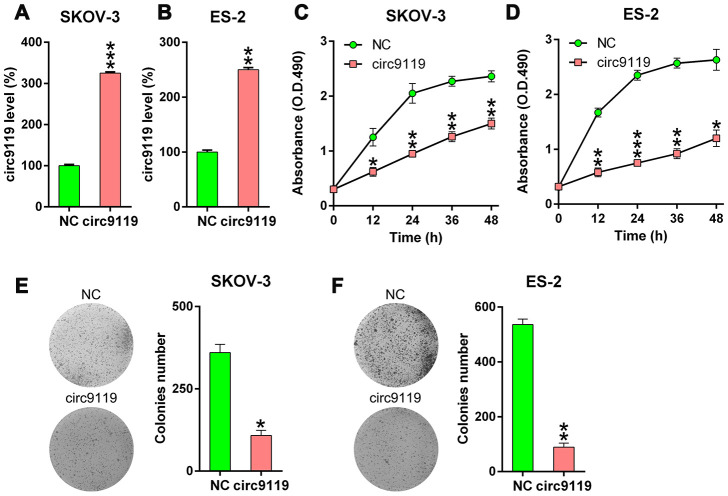
**circ9119 decreased cell viability of ES-2 and SKOV-3 cells.** The cells were transfected with circ9119 overexpression or NC vector for 1.5 d. (**A**, **B**) qRT–PCR examined the circ9119expression. (**C**, **D**) MTT assay showed the proliferation rate of ES-2 and SKOV-3 cells at 12–48 h post transfection. (**E**, **F**) Soft agar colony formation assay conducted in SKOV-3 and ES-2 cells with circ9119 overexpression at 48 h post transfection. n = 3. *P < 0.05, **P < 0.01, ***P < 0.001 vs. the NC groups.

The role of circ9119 in the cell viability of ES-2 and SKOV-3 was studied. circ9119 expression was increased after the cells were transfected the circ9119 overexpressing vector or NC vector (Figure 2A, 2B). The proliferation rates were significantly decreased at 12–48 h after circ9119 overexpression, as indicated by the MTT (3-[4,5-dimethylthiazol-2-yl]-2,5 diphenyl tetrazolium bromide) assay (Figure 2C, 2D). Colony formation assays demonstrated that circ9119 overexpression significantly reduces the number of colonies formed by SKOV-3 and ES-2 cells (Figure 2E, 2F). We also checked the effect of circ9119 knockdown. But actually, there was no obvious effect of circ9119 knockdown on the cell proliferation of SKOV-3 and ES-2 (Supplementary Figure 1).

### circ9119 overexpression triggered cell apoptosis of ES-2 and SKOV-3 cells

**Figure 3:**
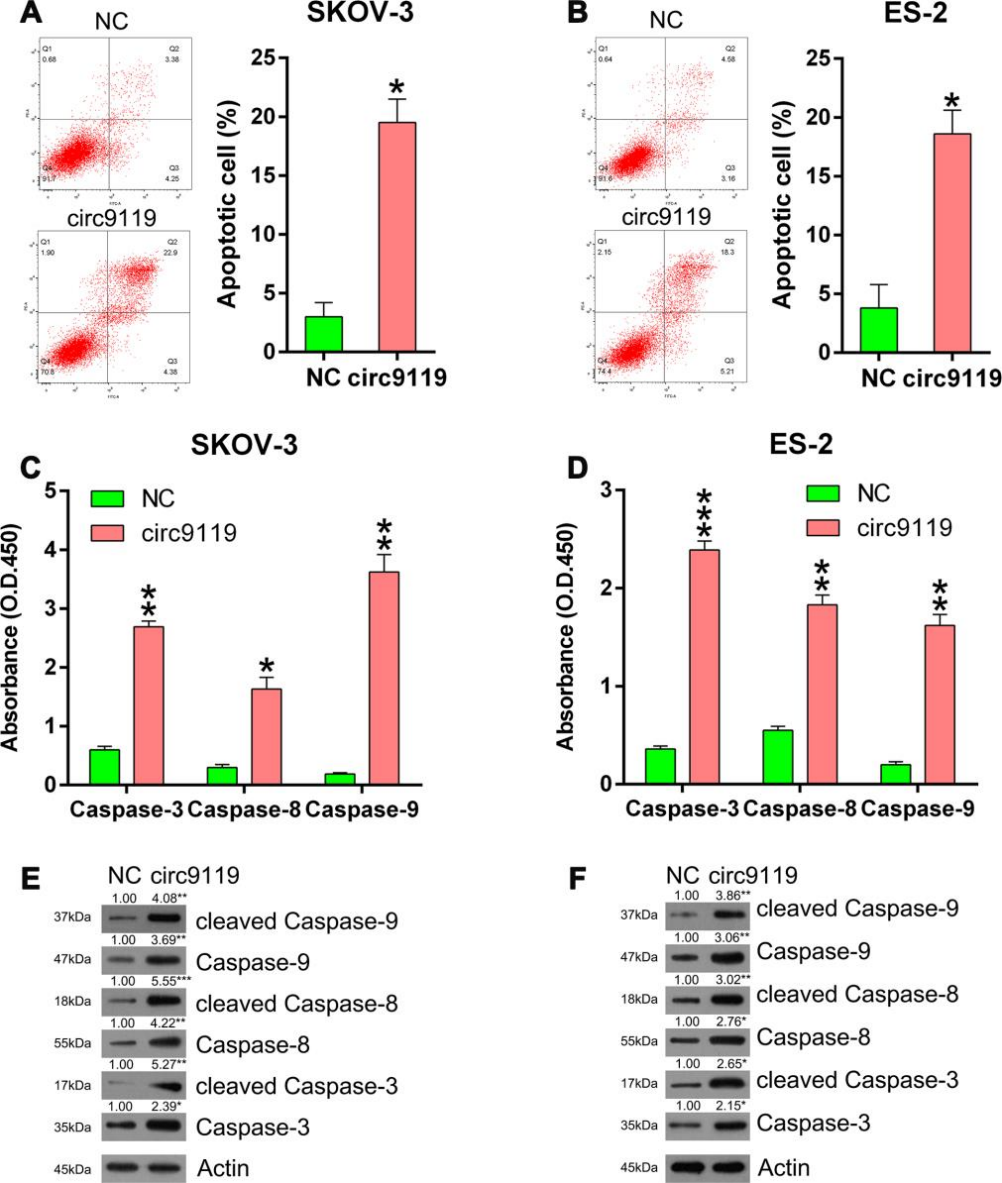
**circ9119 induced apoptosis in ES-2 and SKOV-3 cells.** Cells were subjected to transfection with circ9119 overexpression or NC vector for 1.5 d. (**A**, **B**) Flow cytometry results displayed the number of apoptotic cells. (**C**, **D**) Caspases activity detection kit was used to detect the activity of caspase 3, caspase 8, and caspase 9 post transfection. (**E**, **F**) Western blotting was conducted to examine the expression of caspase 3, caspase 8, and caspase 9 and their cleavage forms. Actin represents β-actin. n = 3. *P < 0.05, **P < 0.01, ***P < 0.001 vs. the NC groups.

We concluded that circ9119 might mediate the cell apoptosis of the six OC cell lines. Compared with the NC groups, annexin V/propidium iodide flow cytometry showed an obvious increase in the number of apoptotic cells of ES-2 and SKOV-3 with circ9119 overexpression (Figure 3A, 3B). Thereafter, we evaluated the activities, expression, and cleavage of caspase 3, caspase 8, and caspase 9, which are typical biomarkers of apoptosis, in ES-2 and SKOV-3 cells. We found that these factors were significantly increased via circ9119 upregulation (Figure 3C–3F), suggesting that circ9119 induced apoptosis of ES-2 and SKOV-3 cells.

### Oncogenic miR-21 is a target of circ9119

**Figure 4:**
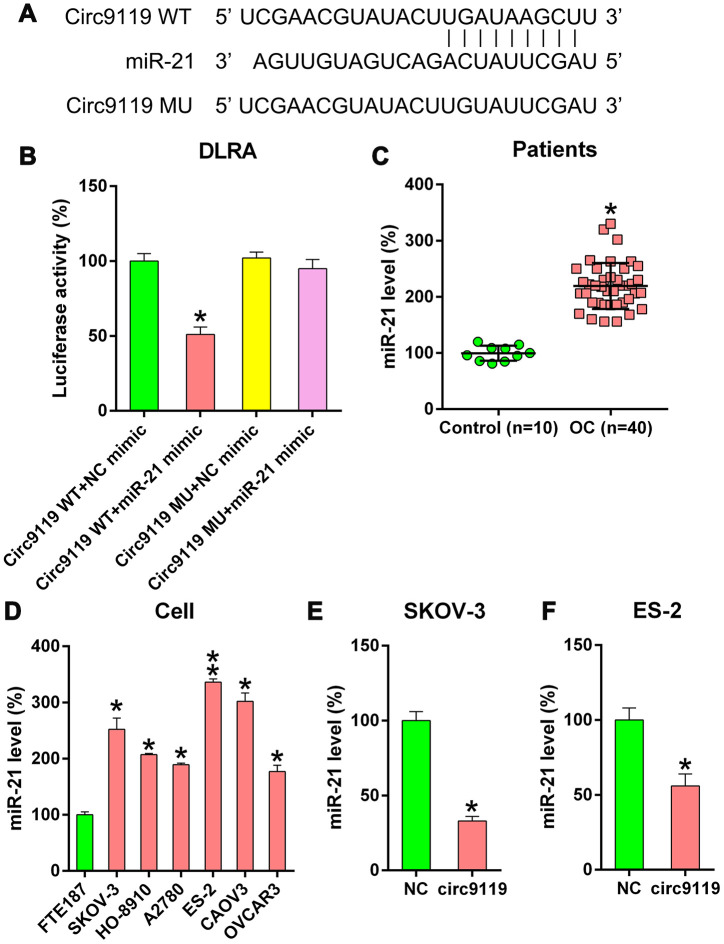
**circ9119 targets miR-21.** (**A**) Bioinformatics forecasting showed an miR-21 binding site in the circ9119 RNA sequence. (**B**) DLRA was conducted and a luciferase reporter containing WT or mutant circ9119 and the miR-21 mimic were subsequently cotransfected into HEK293T cells. (**C**) qRT–PCR displayed the miR-21 expression in HCC samples (n = 40) and paired adjacent normal healthy control samples (n = 10). (**D**) qRT–PCR displayed miR-21 expression in FTE187 and OC cell lines. (**E**, **F**) ES-2 and SKOV-3 cells were transfected with circ9119 overexpressing or NC vector. qRT–PCR examined miR-21 expression. n = 3. *P < 0.05, **P < 0.01 vs. specific groups.

Previous studies have reported the oncogenic role of miR-21 in OC and several other types of cancers [7–9]. In the present study, bioinformatics forecasting tools indicated that circ9119 gene covered the miR-21 seed region-complementary presumptive binding sites (Figure 4A). DLRA was then used to confirm the direct association between miR-21 and circ9119 (Figure 4B). Luciferase activity was found to be reduced by 50% in the HEK293T cells that were cotransfected with WT circ9119 and miR-21 mimic.

Therefore, we determined that circ9119 also served as an miR-21 sponge in the OC samples and cell lines. The miR-21 expression in OC samples and cells was detected. qRT–PCR demonstrated miR-21 upregulation in OC samples compared with that in control samples (Figure 4C). Moreover, increased miR-21 expression was observed in hepatocellular carcinoma (HCC) cells (Figure 4D). We also observed that circ9119 overexpression resulted in a significant decrease in miR-21 expression in the ES-2 and SKOV-3 cells compared with that in the NC cells (Figure 4E, 4F). These data suggest that circ9119 targeted miR-21 in the OC cells.

### miR-21 was involved in circ9119-mediated proliferation of SKOV-3 and ES-2 cells

**Figure 5:**
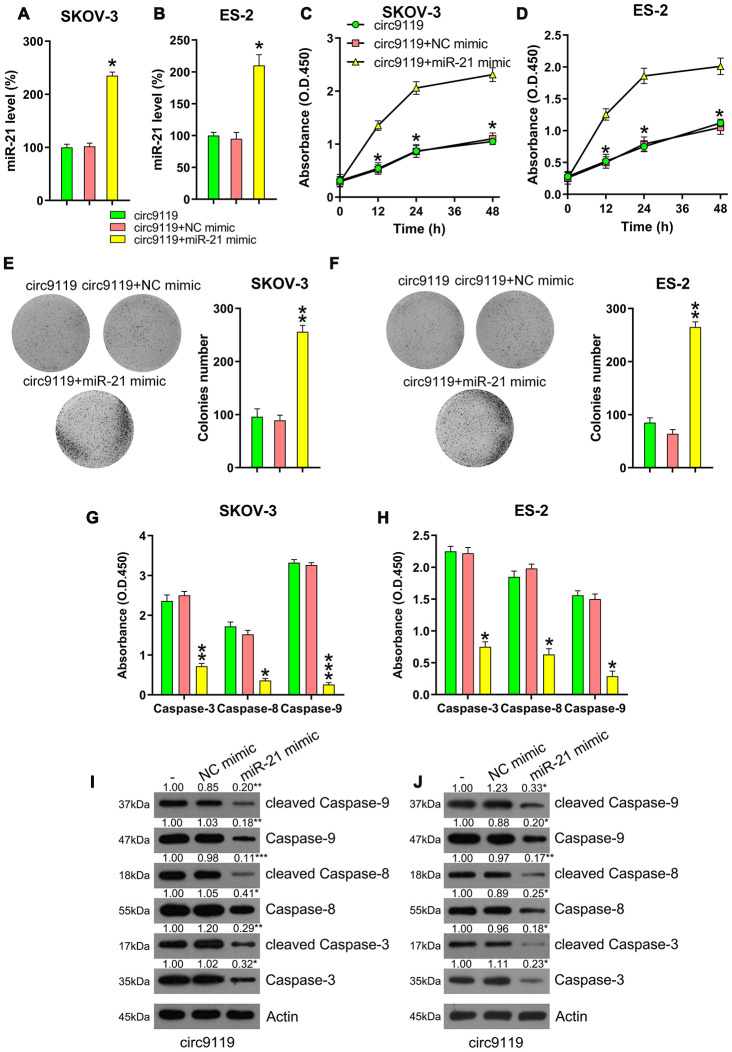
**miR-21 mimic reversed the role of circ9119 overexpression on viability and apoptosis of ES-2 and SKOV-3.** Cells were subjected to cotransfection with circ9119 overexpressing and miR-21 or NC mimic for 1.5 d. (**A**, **B**) qRT–PCR was examined the miR-21 expression. (**C**, **D**) MTT was conducted to detect the proliferation rate of SKOV-3 and ES-2 cells under different transfection conditions. (**E**, **F**) Colony formation assay indicated the growth of ES-2 and SKOV-3 cells under various transfection conditions. (**G**, **H**) Caspase activity detection kit was used to detect the activity of caspase 3, caspase 8, and caspase 9 after transfection. (**I**, **J**) Western blotting was conducted to examine the caspase-3, -8, and -9 expression and their cleavage form. Actin represents β-actin. n = 3. *P < 0.05, **P < 0.01, ***P < 0.001 vs. specific groups.

Several studies have demonstrated that miR-21 can suppress apoptosis in various cancers [10]. Therefore, we hypothesized that miR-21 is involved in the circ9119-mediated proliferation of OC cells. To elaborate the role of miR-21 in cell proliferation, the cells were subjected to cotransfection with a circ9119 overexpressing or NC vector and an miR-21 or NC mimic. qRT–PCR data first demonstrated that miR-21 expression was significantly increased in the circ9119 overexpressing transfection group compared with that in the NC mimic transfection groups (Figure 5A, 5B). The MTT and colony formation assays demonstrated that miR-21 mimics restored the viability and proliferation of circ9119-overexpressing OC cells (Figure 5C–5F). Moreover, caspase activity assay and western blotting showed that miR-21 upregulation caused an obvious decrease in the activities, expression, and cleavage of the caspases, counteracting the effects of circ9119 (Figure 5G–5J). Thus, these data suggest that circ9119 influenced cell viability and apoptosis via miR-21 regulation.

### PTEN–Akt pathway is responsible for circ9119-miR-21-mediated OC cell viability

**Figure 6:**
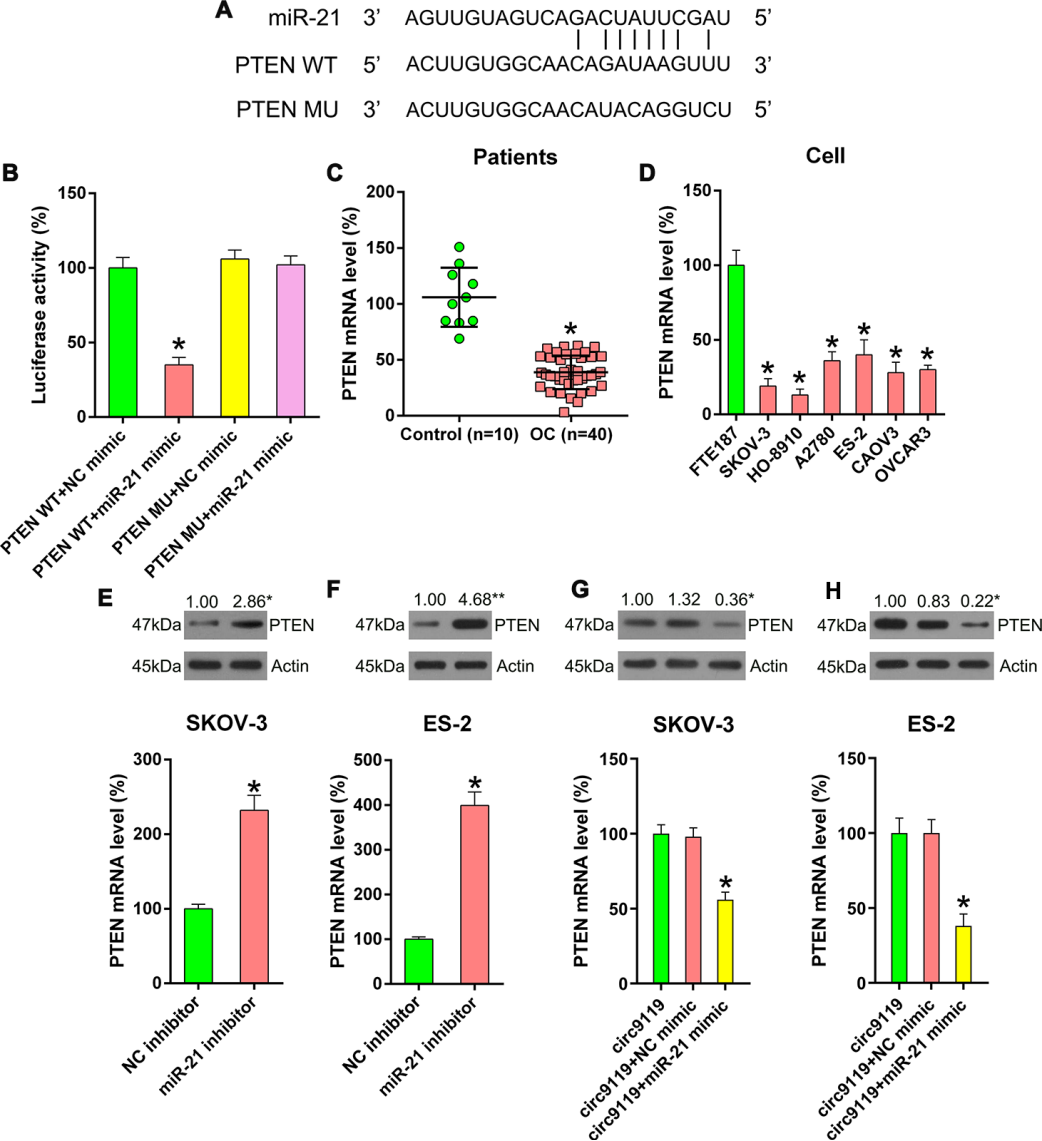
**miR-21 targets the 3′-UTR of PTEN mRNA.** (**A**) Bioinformatics forecasting displayed that miR-21 possesses a binding site in PTEN 3’-UTR. (**B**) DLRA was conducted and a luciferase reporter containing WT or MU of PTEN mRNA and miR-21/NC mimic were cotransfected into HEK293T cells. (**C**) qRT–PCR showed PTEN mRNA expression levels in patients with OC (n = 40) and healthy controls (n = 10). (**D**) qRT–PCR showed the expression levels of PTEN in FTE187 and OC cell lines. (**E**, **F**) ES-2 and SKOV-3 cells were transfected with an miR-21 or NC inhibitor. qRT–PCR and Western blotting were performed to examine PTEN levels. (**G**, **H**) SKOV-3 and ES-2 were subjected to cotransfection with circ9119 overexpressing vector and miR-21 or NC mimic. qRT–PCR and western blotting was performed to examine PTEN expression. Actin represents β-actin. n = 3. *P < 0.05 vs. specific groups.

Several previous studies have demonstrated the direction relationship between miR-21 and the PTEN sensor [11–13]. Here we used bioinformatics forecasting to determine the presumptive miR-21 target on *PTEN*, and the search data displayed that miR-21 possessed binding sites on the 𲀲-UTR of *PTEN* (Figure 6A). DLRA also demonstrated that *PTEN* was a crucial miR-21 objective gene and that transfection with miR-21 mimic can inhibit the decrease in luciferase activity through WT PTEN (Figure 6B). We then analyzed the level of PTEN in the OC samples and cell lines. qRT–PCR revealed an obvious reduction in the mRNA expression of PTEN in the OC samples and cell lines (Figure 6C, 6D).

To thoroughly demonstrate the relevance among the expression of circ9119, miR-21, and PTEN, the cells were transfected with an miR-21 or NC inhibitor to determine PTEN expression. We found that miR-21 inhibition resulted in an increase in PTEN expression (Figure 6E, 6F). Thereafter, the cells were cotransfected with a circ9119 vector and an miR-21 mimic (or NC mimic). Then, qRT–PCR and western blotting showed that the miR-21 mimic decreased the PTEN expression in cells with circ9119 overexpression (Figure 6G, 6H). The findings suggest that the negative correlation between miR-21 and PTEN expression.

**Figure 7:**
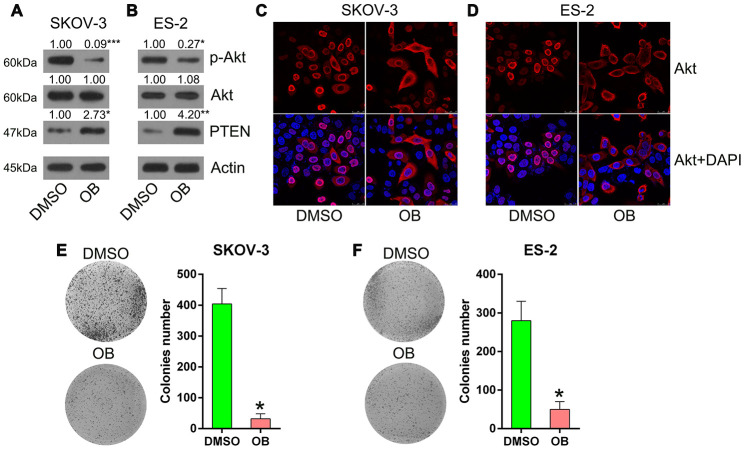
**OB treatment inhibited the growth of SKOV-3 and ES-2 cells. Cells were incubated with 5 μmol/L of OB or 1% of DMSO for 12 h.** (**A**, **B**) Western blotting was used to detect PTEN and Akt expressions as well as the phosphorylated Akt level. Actin represents β-actin. (**C**, **D**) Indirect immunofluorescence assay was conducted to observe the localization of Akt. (**E**, **F**) Colony formation assay indicated the growth of ES-2 and SKOV-3 cells with OB treatment. n = 3. *P < 0.05 vs. specific groups.

To confirm the effects of PTEN on the viability of SKOV-3 and ES-2 cells, these cells were incubated with 5 μM OB (PTEN agonist) or 1% dimethyl sulfoxide (DMSO) for 12 h. Thereafter, the colony formation assay was performed. First, we confirmed the upregulation of PTEN expression after OB treatment and then found that OB treatment inhibited the downstream expression and phosphorylation of Akt was inhibited in the SKOV-3 and ES-2 cells (Figure 7A, 7B). In addition, we observed that the nuclear location of Akt was transported into the cytoplasm on OB incubation (Figure 7C, 7D), indicating that the PTEN pathway was upregulated by OB to block Akt activation. Finally, the colony formation assay showed that OB treatment reduced the number of colonies formed by SKOV-3 and ES-2 cells (Figure 7E, 7F). These data show that OB impaired the growth of OC cells and that PTEN served as a tumor suppressor in OC.

### circ9119 suppressed OC cell proliferation in vivo

**Figure 8:**
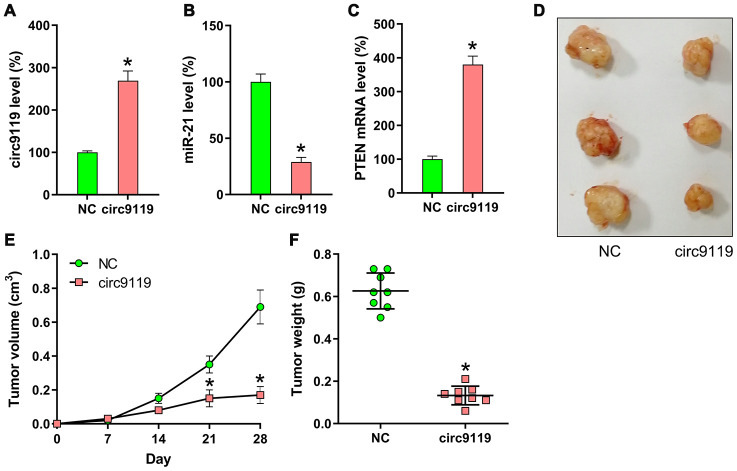
**Xenograft pancreatic cancer formation is suppressed by circ9119 in mice.** SKOV-3 cells transfected with lentiviral-circ9119 or NC cells were administered to the mice subcutaneously (n = 8 in each group). At day 30 post inoculation, we euthanized the animals and weighed the tumors. (**A**) circ9119, (**B**) miR-21, and (**C**) PTEN mRNA expression in OC cells was measured in each group using qRT–PCR. (**D**) three representative images of tumors in each group were displayed. (**E**) Tumor development curve at 27 days post inoculation. (**F**) Tumors from each group were weighed following excision. *P < 0.05 vs. the NC group.

To determine the effects of circ9119 on tumor growth in an in vivo xenograft tumor model, SKOV-3 cells with stably expressing circ9119 or NC were subcutaneously injected into nude mice. The mice were euthanized at 30 days following injection, and their tumors were removed and weighed. circ9119, miR-21, and PTEN mRNA expression in tumors was compared in the two groups (circ9119 group and NC group) at 30 days following injection. Upregulation of circ9119 and PTEN mRNA, and miR-21 downregulation were confirmed in the circ9119 group by qRT–PCR (Figure 8A–8C). The results indicated average volume and weight of the tumors: the circ9119-expressing tumors developed at a much slower rate than the tumors of the mice from the NC group, and the average tumor volume and weight was remarkably lower than those of the tumors in the mice from the NC group at day 28 post injection (Figure 8D–8F).

## DISCUSSION

In the present study, we found that circ9119 was downregulated in the OC samples and cell lines compared with that in the normal healthy control samples. Further, circ9119 overexpression abrogated the proliferation and viability of ES-2 and SKOV-3 cells and induced cell apoptosis. circ9119 might serve as an miR-21 sponge and regulate the PTEN–Akt pathway. The present study has proposed a new mechanism underlying the effect of circ9119 in OC. However, further studies are warranted to evaluate whether cicr9119 is associated with several clinicopathological variables or is correlated with poor patient outcomes.

circRNAs play a crucial role as a gene regulator by regulating gene and protein expressions, and they are involved in various physiological functions and pathological reactions [14]. Some circRNAs have been identified to exert crucial regulatory effects as miRNA sponges [15]. Many relevant studies have focused on different types of cancers, including OC. An increasing number of studies have reported the crucial role of circ9119 in several diseases. For example, the involvement of circ9119 has been reported in receptive endometrium (RE) and testicular inflammation [3, 16]. In the RE of dairy goats, increased expression of circ9119 and PTGS2 and decreased expression of miR-26a were detected using qRT–PCR. circ9119 was able to reduce the miR-26a expression by serving as an miRNA sponge and miR-26a was found to downregulate PTGS2 expression through the presumptive target site in the endometrial epithelial cells of dairy goats in vitro [3]. Another study found elevated expression of TLR3, circ9119, and RIG-I and reduced expression of miR-26a and miR-136. Decreased circ9119 expression was found to inhibit the inflammatory responses in poly I:C-treated Sertoli and Leydig cells. circ9119, as an miRNA sponge, suppressed the expressions of miR-26a and miR-136. The decreased expression of miR-26a and miR-136 recovered the expression of inflammatory cytokines to some extent that had been suppressed on circ9119 silencing [16]. After examining the findings of this previous study and the present study, we found that the circ9119 sequence is not conserved between murine and human cells. The present study found that circ9119 acted as an miR-21 sponge and was involved in the proliferation, viability, and apoptosis of OC cells. The overexpression of circ9119 suppressed the viability of ES-2 and SKOV-3 cells and induced cell apoptosis. The bioinformatics analysis and DRLA showed that circ9119 served as an miR-21 sponge; moreover, its upregulation in the OC cell lines resulted in an increased expression of PTEN and a decreased expression of miR-21. However, after surveying previous studies, we found that circ9119 sequence is not conserved between rodents and human [3, 16]. This may block the application of circ9119 in other similar research with different species.

The deletion of PTEN on chromosome 10 has shown to negatively mediate the phosphatidylinositol-3 kinase (PI3K) and protein kinase (Akt) pathway and play a critical role in the regulation of cell proliferation, migration, invasion, and apoptosis [17]. Decreased PTEN expression has been reported to be associated with the pathogenesis of some types of cancers, such as gastric [18], lung [19], and thyroid cancers [20]. Previous studies have demonstrated a relationship between the ectopic high expression of miR-21 and the development, progression, and drug resistance of OC, suggesting that miR-21 serves as a cancer suppressor gene in OC [7, 21]. The PTEN–Akt pathway was implicated in the occurrence of various cancers, such as breast carcinoma and gastric cancer [22–25]. The data in our study suggested that circ9119 regulates the PTEN–Akt pathway by decreasing the upstream expression of miR-21. These observations revealed that circ9119 is a positive modulator, whereas miR-21 is an essential negative modulator. Using qRT–PCR, we observed that the endogenous levels of circ9119 in the OC cell lines reduced substantially compared with those in the normal FTE187 cells. Conversely, the expression of miR-21 in the OC cells was obviously augmented, leading to a considerable reduction in the expression of PTEN, thus affecting Akt phosphorylation and nuclear localization.

In conclusion, the present study demonstrated that circ9119 was a tumor suppressor that inhibited the cell viability of OC via competitive interaction with miR-21, subsequently influencing the PTEN–Akt pathway. This study provides a mechanistic understanding of the role of circ9119 in OC and suggests that circ9119, JAK1, or STAT3 serves as prognostic factors and potential therapeutic targets in OC treatment.

## MATERIALS AND METHODS

### Clinical samples

Samples from 40 patients with OC (OC samples) were collected from the Obstetrics and Gynecology Department of the First Affiliated Hospital of Wenzhou Medical University. The clinical characteristics of these patients are summarized in Table 1. In addition, fresh normal ovarian tissues were each collected from 10 patients (normal healthy control tissues). All samples were frozen and stored in liquid nitrogen until further analysis. All specimens had been confirmed via pathological diagnosis. This study was approved by the Institutional Research Ethics Committee of the First Affiliated Hospital of Wenzhou Medical University. All the patients participated in the study from January 2015 to January 2019 and provided written informed consent.

**Table 1:** Clinical characteristics of study patients.

**Characteristics**	**Number of patients (n = 40)**
Age (years)	
>50	15
≤50	25
Ascites	
>100	13
≤100	27
Serum CA-125	
>35	5
≤35	35
Tumor size	
>3 cm	33
≤3 cm	7
Lymph node metastasis	
Negative	23
Positive	17
FIGO stage	
I–II	8
III–IV	32

FIGO: International Federation of Gynecology and Obstetrics.

### Cell culture and transfection

A total of six OC cell lines—SKOV-3, HO-8910, A2780, ES-2, CAOV3, and OVCAR3—and FTE187 (an immortalized human fallopian tube epithelial cell line) were purchased from the American Type Culture Collection (ATCC; Manassas, VA, USA). OC cells were cultivated in Dulbecco's modified Eagle's medium supplemented with 10% fetal bovine serum (FBS) and 0.1 g/mL of penicillin–streptomycin. FTE187 cells were cultivated in medium 199 and MCDB105 medium mixed at a ratio of 1:1 supplemented with 10 ng/mL of epidermal growth factor solution and 10% FBS. All these cells were preserved in a humidified incubator with 5% CO_2_ atmosphere at 37°C. Before further examination, FTE187 cell were cultured in the same medium as other OC cells for three generations to remove the interference of culture medium factors.

miR-21 mimics and inhibitors and the corresponding negative control (NC) vectors were obtained from Ribobio (Guangzhou, China). The circ9119 overexpressing vectors—pcD-ciR-circ9119 and pcD-ciR-NC—were constructed in our laboratory. All the aforementioned vectors were transfected into OC cells using Lipofectamine 2000 (Invitrogen, Carlsbad, CA, USA).

### Establishment of circ9119 overexpressing plasmids and lentiviral-circ9119 particles

To establish circ9119 overexpressing plasmids, human circ9119 cDNA was prepared and cloned into pcD-ciR vectors that comprised a front and back circular frame. The resulting plasmids were transfected into cells using Lipofectamine 2000.

The circ9119 oligonucleotide scrambled and NC sequence were phosphorylated, annealed, and cloned into the pLVX-puro vector (Clontech Laboratories, California, USA), designated as pLVX-circ9119 and pLVX-NC. Lentiviral-circ9119 and lentiviral-NC particles were produced by triple transfection of 293T cells (Invitrogen) using the vectors pLVX-circ9119 and pLVX-NC, respectively, along with psPAX2 and pMD2.G, respectively. For infection studies, SKOV-3 cells were incubated with lentiviral particles and polybrene (5 μg/mL) in a growth medium. After 6 h, the infection medium was discarded and the cells were used for analysis.

### Microarray and quantitative evaluation

The specimens used in the microarray were randomly selected from the 40 OC samples and 10 normal healthy control tissues described above. OC tissues were shock-frozen at once using liquid nitrogen. The specimens (three poly I:C-supplemented and three non-supplemented) were homogenized with TRIzol reagent (Invitrogen). NanoDrop ND-1000 was used to quantify total RNA in all the samples. The total RNA extracted from all the samples was amplified and transcribed to fluorescent circRNA using random primers and the Super RNA Labeling Kit (Arraystar Inc., Rockville, USA), according to the manufacturer’s instruction. Arraystar Human Circular RNA Microarray was used to hybridize the labeled circRNAs. Agilent G2505C Scanner (Agilent Technologies, California, USA) was used to scan arrays after washing the slides.

### Quantitative reverse transcription–polymerase chain reaction (qRT–PCR)

Total RNA was extracted using TRIzol. RNA solution supplemented with RNase-free DNase 1 was used to remove DNA contaminants. The resulting mixture was examined with PCR; U6 and β-actin were used as an internal control for miR-21 and other mRNA, respectively. PCR was conducted using the Green PCR Master Mix kit on a real-time cycler, and the 2^−ΔΔCT^ method [26] was used to quantify the relative transcript expression levels. The primer sequences were as follows: circ9119 forward 5′-GAA TGG GAT TCG AGA CCT G-3′, reverse 5′-TTC TTC CAA AGC TGC CTG T-3′; miR-21 forward 5′-ACA CTC CAG CTG GGT AGC TTA TCA GAC TGA-3′, reverse 5′-TGG TGT CGT GGA GTC G-3′; β-actin forward 5′-GTG GCC GAG GAC TTT GAT TG-3′, reverse 5′-CCT GTA ACA ACG CAT CTC ATA TT-3′; U6 forward 5′-CTC GCT TCG GCA GCA CA-3′, reverse 5′-AAC GCT TCA CGA ATT TGC GT-3′; PTEN forward 5′-CGG CAG CAT CAA ATG TTT CAG-3′, reverse 5′-AAC TGG CAG GTA GAA GGC AAC TC-3′.

### Western blotting

Proteins (15–20 μg/well) were isolated on 10% sodium dodecyl sulfate and polyacrylamide gels, and the obtained bands were electronically transferred onto polyvinylidene fluoride membranes, followed by blockage with Tris-buffered saline (TBS) and 5% skimmed milk at room temperature for 60 min. The membranes were incubated at 4°C with primary antibodies: anti-PTEN (1:1000, SAB1406331, Sigma), anti-phospho-Akt (1:500, SAB4504331, Sigma), anti-Akt (1:2000, SAB4500797, Sigma), and anti-Actin (1:5000, A2066, Sigma) antibodies. The membranes were washed twice with TBS, to which 0.1% Tween-20 was added prior to incubation with secondary antibodies. The Ag/Ab complex was examined using the enhanced chemiluminescence detection kit with β-actin as the loading control.

### Flow cytometry

The cells were collected using trypsin digestion without EDTA and then centrifuged at room temperature at 1,000 g for 5 min. Subsequently, the cells were re-suspended in 300 μl of 1 X binding buffer (BD Pharmingen™). A total of 5 μl of Annexin V-FITC (BD Pharmingen™) was the added to the samples and mixed for 15 min at room temperature in the dark. Subsequently, 5 μl of propidium iodide (BD Pharmingen™) was added for 5 min. The samples were analyzed using a flow cytometer (FACSCalibur; BD Pharmingen™). FlowJo software (version 7.6.1; FlowJo LLC) was used to analyze the data.

### Indirect immunofluorescence assay

The OC cells were fixed with polyoxymethylene at 25°C for 0.5 h and then permeabilized with 0.2% Triton X-100 in phosphate-buffered saline (PBS) for 15 min. The cells were blocked with 5% common goat serum, incubated with primary antibodies, washed with PBS twice, and incubated again for 60 min with proper secondary antibodies bound to fluorescein isothiocyanate/tetramethylrhodamine isothiocyanate. The cells were then mounted with neutral balsam and observed under Leica SP8 microscope.

### Dual luciferase reporter assay (DLRA)

DLRA was conducted to examine the miR-21 objective genes. Luminescence was calibrated with the firefly luciferase sequence as the basis. *Renilla* luciferase served as a reporter. The cells were co-transfected with miRNA mimics or NCs along with luminescence vectors—wild-type (WT) or mutant PTEN 3′ untranslated region (UTR) and circRNA-9119—and then incubated 1.5 d. Thereafter, the luciferase activities were evaluated.

### PTEN agonist treatment

Oroxin B (OB), a PTEN agonist (SMB00340, Sigma), was used to promote PTEN activity and inhibit Akt phosphorylation [27]. The cells were incubated with 5 μM OB for 12 h.

### Bioinformatics prediction

We used the prediction algorithm starBase (http://starbase.sysu.edu.cn/) and TargetScan (http://www.targetscan.org) to identify the targets of circ9119 and miR-21, respectively. Predictions are listed according to the prediction of targeting efficacy. As an alternative, predictions are also ranked by their probability of conserved targeting [28].

### Animal experiments

BALB/c nude mice (5-week old, female) were purchased from Vital River (Beijing, China). SKOV-3 cells (1 × 10^6^) were infected with lentiviral-circ9119 or lentiviral-NC particles to generate SKOV-3 cells with constitutive expression of circ9119 or NC, respectively. All mice received a subcutaneous injection of transfected cells in the right armpit after adapting to the environment for 3 d. Tumor weights are presented in grams, and the formula (L × W2)/2 was used to measure tumor size. On the 30^th^ day following injection, the mice were euthanized.

### Caspase activity assay

Caspase activity was determined using colorimetric assay kits with p-nitroaniline (pNA)-labeled synthetic tetrapeptides (Asp–Glu–Val–Asp for caspase 3, Leu–Glu–His–Asp for caspase 9, and Ile–Glu–Thr–Asp for caspase 8). Briefly, the cells treated with or without deoxyazacytidine were lysed in a lysis buffer and centrifuged. The supernatant was collected and incubated with reaction buffer at 37 °C. Changes in the absorbance were read at 405 nm with a VERSAmax tunable microplate reader.

### Statistical analysis

Data are presented as average ± standard deviation values. ANOVA and t-test were applied to compare the groups. P values of <0.05 were considered statistically significant.

## Supplementary Material

Supplementary Figure 1
