# Supplementary material for: Circular RNA-9119 suppresses in ovarian cancer cell viability via targeting the microRNA-21-5p–PTEN–Akt pathway
Source: Aging (Albany NY). 2020 Jul 16;12(14):14314–28. doi: 10.18632/aging.103470 (PMC7425477; doi:10.18632/aging.103470)
Supplement: Supplementary Figure 1 [file aging-12-103470-s001..pdf]

SUPPLEMENTARY FIGURE

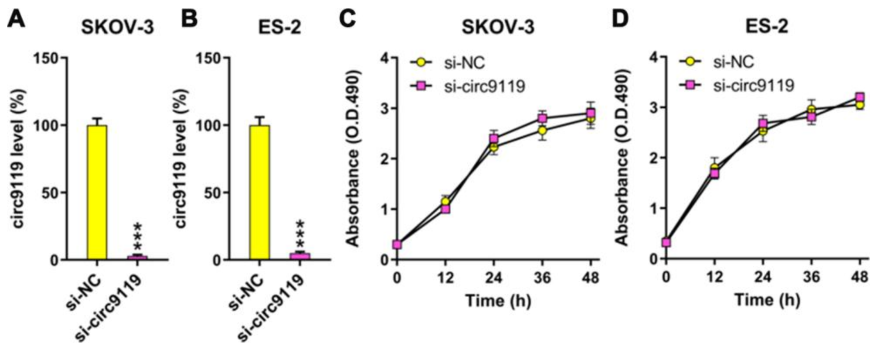

Supplementary Figure 1. Role of circ9119 silencing on the proliferation of SKOV-3 and ES-2 cells. (**A**, **B**) SKOV-3 and ES-2 cells were transfected with siRNA-NC or siRNA-circ9119. qRT–PCR examined the circ9119 expression. (**C**, **D**) MTT assay showed the proliferation rate of SKOV-3 and ES-2 cells at 12–48h post transfection.
